# A cross-sectional analysis of gender and psychological well-being among older Taiwanese adults

**DOI:** 10.3389/fpsyg.2024.1392007

**Published:** 2024-06-18

**Authors:** Deleon N. Fergus, Yi-Hua Chen, Ying-Chih Chuang, Ai-hsuan Sandra Ma, Kun-Yang Chuang

**Affiliations:** ^1^School of Public Health, Taipei Medical University, Taipei, Taiwan; ^2^Department of Sociology, National Chengchi University, Taipei, Taiwan

**Keywords:** psychological well-being, gender, health, aging, culture, socioeconomic, Taiwan

## Abstract

**Background:**

Psychological well-being (PWB) facilitates good health. Few studies have taken into consideration gender and how it can affect PWB within a sociocultural context. This study aims to determine if relationships between social, health, behavioral, and socioeconomic factors on PWB among older Taiwanese adults are affected by gender.

**Methods:**

Data were obtained from the 2016 Taiwan Mental Health Survey. A representative sample, of 2,286 individuals, was created using multistage proportional probability. Participants were interviewed at their homes using a structured questionnaire. Inclusion criteria were Taiwanese citizenship, age ≥ 55 years, and the ability to provide informed consent. Participants 65 years and above were selected for the study sample *n* = 1,533. An 18-item version of Ryff’s PWB scale was used to determine PWB. The median value was used to categorize low and high PWB. Logistic regression analyses were used to examine predictors of PWB stratified by gender.

**Results:**

Chronic disease, unemployment, and financial dependence negatively impacted men’s PWB. Satisfaction with living environment and family relationships positively impacted women’s PWB. Unique characteristics of older men, women, and culture account for this.

**Conclusion:**

Gender-specific interventions aimed at promoting PWB in older adults are needed. Recommendations include educational programs, social support workshops, and community engagement initiatives.

## Introduction

Population aging is a global phenomenon (Benziger et al., 2016) with profound implications for public mental health and well-being. As the world ages, the prevalences of mental distress such as depression, anxiety, and general poor mental health, are expected to increase (Hallit et al., 2020). Poor mental health significantly affects an older adult’s ability to perform activities associated with everyday life (de Mendonça Lima et al., 2019). Therefore promoting good mental health, particularly among older adults is a public health concern, especially in rapidly aging populations such as Taiwan (Lin and Huang, 2015).

Psychological well-being (PWB) is believed to provide a buffer against mental health problems (Ryff and Singer, 1998). It is defined as a feeling of happiness and contentment while functioning effectively and productively (Huppert, 2009). Individuals with low and high PWB are, respectively, described as flourishers and languishers. Flourishers are often productively engaged in society. On the other hand, languishers live a life of quiet melancholy and have increased risks of depression and cardiovascular diseases (Huppert, 2009).

This study uses a eudemonic framework to assess psychological well-being, Ryff’s model. In this approach, psychological well-being involves the pursuit of happiness by finding meaning, purpose, and self-fulfillment (Ryff and Keyes, 1995). Ryff’s model of psychological well-being is comprehensive, featuring autonomy, personal growth, positive relations with others, purpose in life, environmental mastery, and self-acceptance. Ryff’s model encompasses the flourishing individual and can be used as a tool in examining and improving well-being as an approach to promoting good health (Slade, 2010).

Ryff’s model can be applied practically in assessing and promoting psychological well-being across many disciplines and potentially guide interventions. However, Ryff’s model does not explicitly identify how advanced years and gender differences impact psychological well-being. However, further study of this model suggests that there is some intersectionality with sociodemographic factors, gender, cultural norms, and psychological well-being (Ahrens and Ryff, 2006).

Other studies have found some interesting associations surrounding psychological well-being (PWB). Socioeconomic, health, (Ryff and Singer, 2008), behavioral and social-related factors were found to be predictors of PWB. Socioeconomic factors such as being married (Huppert, 2009; Wright and Brown, 2017), having a higher education (Ahrens and Ryff, 2006; Ryff and Singer, 2008), being employed (Ryff and Singer, 2008), and practicing religion (Bradshaw and Kent, 2017) were found to be associated with higher PWB. However, it is unclear how advanced age or gender impacts these associations. Additionally, metropolitan regions such as big cities have the potential to provide both the best and worst environments for health and well-being (Krefis et al., 2018). Big cities are often rife with heat stress, and air and noise pollution, which are risk factors for reduced well-being (Krefis et al., 2018). On the other hand, cities also provide opportunities for social interactions and participation in social activities which can improve well-being (Krefis et al., 2018). The role advanced age plays in the relationship between place of residence and PWB is unclear. Population aging means increasing numbers of older adults in metropolitan areas.

Better physical health (Hernandez et al., 2018) was generally found to be associated with higher PWB in the literature. It was found that social factors such as relationships with friends (Sharifian and Grühn, 2018) and family (Kalaitzaki et al., 2020) play a key role in the PWB of older adults.

Behavioral factors such as community participation also have notable associations with higher PWB in older adults (Lee et al., 2018; Sapranaviciute-Zabazlajeva et al., 2018). Additionally, the use of the internet, specifically social technology was also found to be associated with better mental health as it circumvents loneliness (Chopik, 2016; Fang et al., 2019). However, it is unclear how gender impacts these associations. Caregiving positively affects PWB as long as it does not become burdensome to the older adult (Tang et al., 2016). However, the role gender plays in the relationship between community participation, caregiving and PWB is unclear.

For this study, gender is discussed as “male” and “female.” It was found that women are more likely to have greater levels of psychological distress (Yu, 2018) but there is little understanding of how the predictors of PWB are differentially affected by gender. This study aims to address these gaps in a crucial segment of the Taiwanese population (older adults), by investigating the role gender plays in influencing the relationships between sociodemographic, health, social, and behavioral factors and PWB.

### Research questions

1. How do socioeconomic, health, social, and behavioral factors predict psychological well-being among older Taiwanese adults?
2. How do gender differences influence the relationships between predictors of psychological well-being and well-being outcomes?

### Hypothesis

There are gender differences in the socioeconomic, health, social, and behavioral predictors of psychological well-being among older Taiwanese adults with some having a stronger association with psychological well-being in women compared to men while others show a stronger association in men.

## Methods

### Design, data, sampling, and study participants

**Figure 1:**
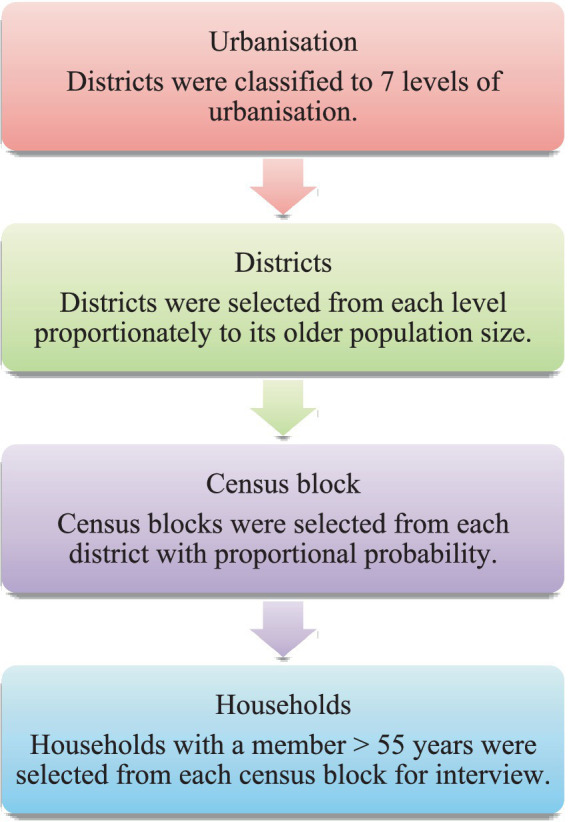
Sampling procedure of prospective survey participants under study.

**Figure 2:**
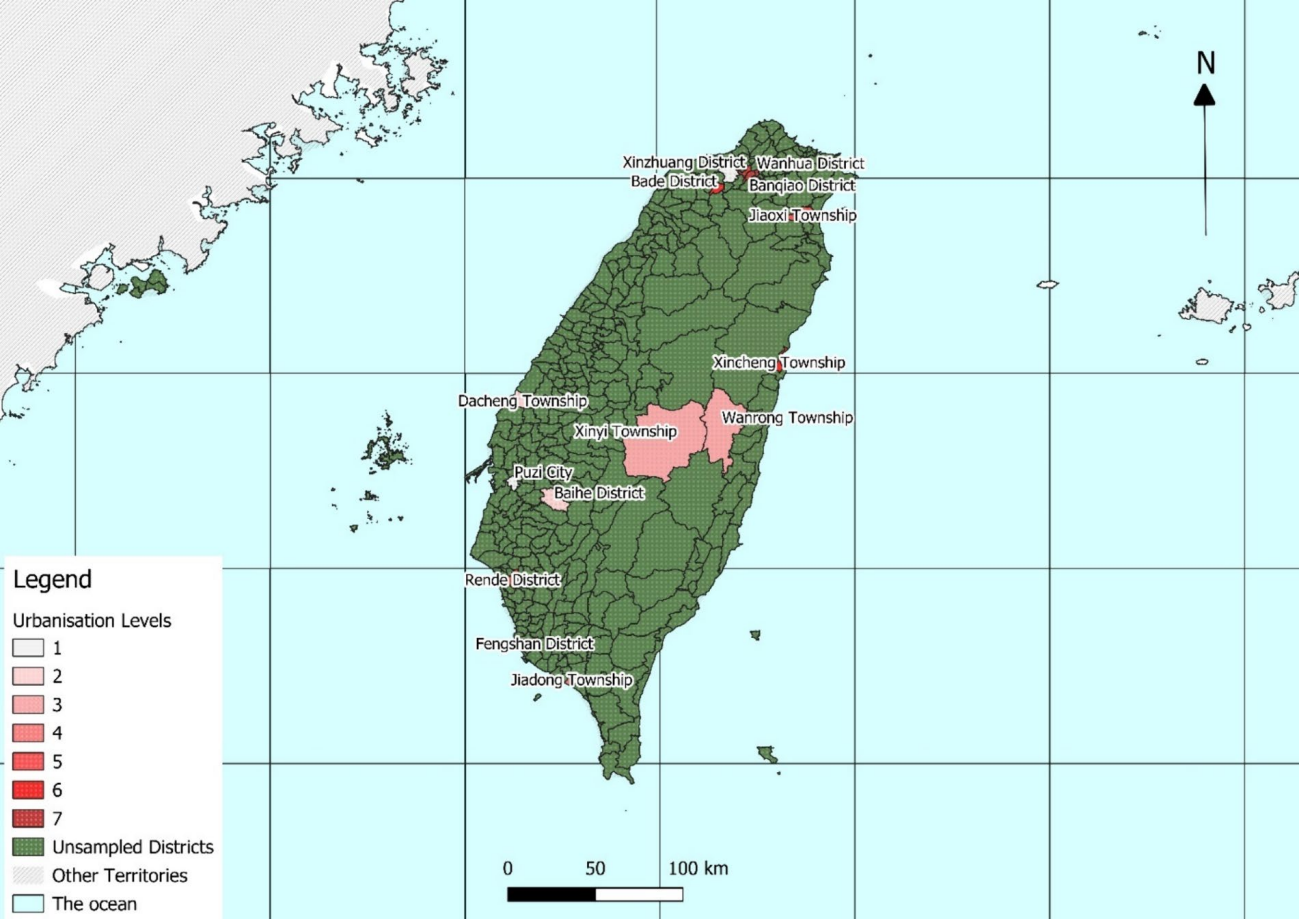
Urbanization levels of select sampled areas in Taiwan.

The dataset used in this research was obtained from A Taiwanese Survey of Mental Health conducted in 2016. The sample size was 2,286 and was representative of the national population. The sample was obtained using the multistage proportional probability to size (Figure 1). The sampling frame consisted of a list containing all districts on the main island which encompasses 98.9% of the entire national population. Each district was categorized into one of seven levels of urbanization (with level 1 being the most urbanized) through a cluster analysis that considered population density, the population over 65 years old, the number of people who had achieved junior college education or higher, the number of Western medical institutions, and the number of farmers and herdsmen. In this study, level 7 was recoded as the highest level of urbanization and level 1 the lowest (Figure 2).

Districts selected from each level with the probability of selection proportional to the size of its older population to minimize data collection bias.

*Level 1* consisted of Guishan District (Taoyuan City) and Puzi City (Chiayi County).

*Level 2* consisted of Baihe District (Tainan City) and Dacheng Township (Changhua County).

*Level 3* consisted of Xinyi Township (Nantou County) and Wanrong Township (Hualien County).

*Level 4* consisted of Yuanli Town (Miaoli County) and Jiadong Township (Pingtung County).

*Level 5* consisted of Minxiong Township (Chiayi County) and Jiaoxi Township (Yilan County).

*Level 6* consisted of Fengshan District (Kaohsiung City), Nantou City (Nantou County), Xincheng Township (Hualien County), Bade District (Taoyuan City), Rende District (Tainan City) and Yilan City (Yilan County).

*Level 7*, the most urbanized, consisted of Banqiao District (New Taipei City), Wanhua District (Taipei City), Central District, (Taichung City), and Xinzhuang District (New Taipei City).

Census blocks in each district were selected using proportional probability according to the population of residents. Households from these census blocks that had at least one individual aged ≥55 years were selected using the government household registry. The household registration addresses were sorted using simple random sampling for each census block.

Interviewers visited the household to verify that there was an eligible participant. Inclusion criteria were Taiwanese citizenship, age ≥ 55 years, and the ability to provide informed consent. The interviewer read from the questionnaire and filled out the responses at the participants’ homes using paper and pencil. This paper only included participants who were ≥ 65 years of age in our study sample thus the study sample featured 1,533 participants.

### Measurements

PWB was measured using an 18-item version of the Carol Ryff Psychological Well-Being Scale (Li, 2014). The sum of 18 scored items (1 ~ 5 strongly disagree, disagree, neutral, agree, and strongly agree) consisted of the PWB score, a combination of the six dimensions of psychological well-being in Ryff’s model. The median score (*M* = 69) was used as a “cutoff” mark (Sapranaviciute-Zabazlajeva et al., 2018). Scores of ≤69 were categorized as “low PWB” and scores of >69 were categorized as “high PWB.”

Predictor variables were classified as socioeconomic, health, social, and behavioral factors. Gender was categorized as “male” or “female.” Age, education, marital status, religious beliefs, employment, financial stress, living environment, and financial dependence were classified as socioeconomic. Religion and employment were dichotomized. Education was described as no education, primary, secondary, and tertiary. Marital status was “married” and “not married (single, divorced, separated, or widowed). Financial stress was assessed by the question, “Do you have difficulty in meeting your household expenses?” Responses of “very difficult” and “with some difficulty” were categorized as high, and “no difficulty at all” and “little difficulty” were categorized as low financial stress. The question, “Overall, how satisfied are you with your living environment?” was used to determine satisfaction with their living environment which was dichotomized into “satisfied” and “dissatisfied.” Place of residence was derived from the seven levels of urbanization during the sampling process. The first level of urbanization consisted of the most urbanized areas in Taiwan and was described as “metropolitan,” while the other six levels were described as “other.” Metropolitan locales included Taipei City, New Taipei City, and Taichung City.

Financial dependence was assessed by the question, “What is your main source of income at present?” Participants who received child support or revenue from the state as their main source of income were considered “dependent,” and those who did not were “not dependent.”

Health factors included self-reported health, chronic diseases, sleep quality, and body pain. The questions “In general, how is your health?” and “How was your sleep in the past week” respectively assessed self-reported health and sleep quality. The original five response categories (very good, good, fair, bad, and very bad) were amalgamated into three (poor, fair, and good). The presence of a chronic disease was determined by asking a participant if a healthcare provider had diagnosed them with any of the following: diabetes, hypertension, hyperlipidemia, duodenal ulcer, or mental illness (depression, anxiety, or dementia). The question “Did you have body pain during the past 4 weeks? If so, how much?” was used to assess the body pain variable. The original six-point scale was amalgamated into the three categories of “none,” “mild to moderate,” and “severe.”

Social factors consisted of family relationships and time spent with friends. The strength of family relationships was measured by three questions: “How frequent is your interaction?,” “How is the quality of the relationship?,” and “How well do you get along with family members?” Each response was allocated a score of 1 ~ 5 (Chiang et al., 2013). A higher score for the three questions indicated a better relationship. A median of 12 was used as a cutoff to create the categories of “low” and “high.” Time spent with friends was assessed by the amount of time participants spent socializing with their friends as “little,” “fair,” and “a lot.”

Behavioral factors included caregiving, participation in community activities, and internet use. The question, “Does anyone in your family need your care due to chronic disease, mental illness, old age, dementia, disability, cancer, major injury, etc.?” to determine if they performed a caregiving role. Community participation was dichotomized (yes or no) and included participation in organizations, religious, charity, or other social functions. Time spent with friends examined the amount of time spent interacting with their friends. Daily internet use was dichotomized as yes or no.

### Data analysis

Data was analyzed using SPSS for Windows version 22 (SPSS, Chicago, IL, USA). The study sample (*N* = 1,533) was created after data cleaning and selecting all participants ≥65 years with complete data fields, and then descriptive characteristics were determined. Bivariate and multivariate logistics analysis were performed with PWB, then stratified by gender using a 95% confidence interval (CI) to report the percentage of the odds ratio (OR).

### Ethical considerations

Each study participant provided written informed consent after the purpose and contents of the survey were explained. This study was approved by the Taipei Medical University Joint Institutional Review Board (no.: 201608036).

## Results

Table 1 shows that the mean age was 74.0 ± 6.8 years with a range of 37. Participants were mainly female (58.8%), married (65.2%), unemployed (85.6%), practiced religion (84.2%) and 47% had primary education at the highest level. Most reported that their financial stress was low (76.2%), they were satisfied with their living environment (72.2%), but they were financially dependent on others (65.7%). Most did not perform a primary caregiving role (84.7%), reported fair health (42.9%) and good quality sleep (44.8%). Although most had been diagnosed with a chronic disease (67.7%) and had experienced mild to moderate body pain (54%). Most of the study sample did not report participation in community activities (60.8%) but spent a fair amount of time with friends (55.5%). Daily internet use was low (16.8%). Most had high family relationship scores (70.8%) and lived outside of metropolitan regions (74%). The average family relationship score was 12.3 ± 1.9 with a range of 12. It was found that 45.9% of the sample had high PWB. The average PWB score was 67.7 ± 9.2 with a range of 70.

**Table 1:** Descriptive characteristics of the study sample.

Variable	*n* (*N* = 1,533)/Mean ± SD	%/Range
*Gender*		
Male	632	41.2
Female	901	58.8
*Marital status*		
Not married	534	34.8
Married	999	65.2
*Employment*		
No	1,313	85.6
Yes	220	14.4
*Educational level*		
None	364	23.7
Primary	720	47.0
Secondary	329	21.5
Tertiary	120	7.8
*Religious beliefs*		
No	242	15.8
Yes	1,291	84.2
*Financial stress*		
High	365	23.8
Low	1,168	76.2
*Satisfaction with the living environment*		
Dissatisfied	426	27.8
Satisfied	1,107	72.2
*Place of residence*		
Metropolitan	390	25.4
Other	1,143	74.6
*Financial dependence*		
Not dependent	526	34.3
Dependent	1,007	65.7
*Caregiver role*		
No	1,299	84.7
Yes	234	15.3
*Self-reported health*		
Poor	366	23.9
Fair	657	42.9
Good	510	33.3
		
*Chronic diseases*		
No	495	32.3
Yes	1,038	67.7
*Sleep quality*		
Poor	411	26.8
Fair	435	28.4
Good	687	44.8
*Body pain*		
None	611	39.9
Mild to moderate	828	54.0
Severe	94	6.1
*Community participation*		
No	932	60.8
Yes	601	39.2
*Time spent with friends*		
Little	574	37.4
Fair	851	55.5
A lot	108	7.0
*Daily internet use*		
No	1,276	83.2
Yes	257	16.8
*Family relationship score*		
Low	447	29.2
High	1,086	70.8
*Psychological well-being*		
Low	829	54.1
High	704	45.9
*Age (years)*	74.0 ± 6.8	37.0
*Family relationship score*	12.3 ± 1.9	12.0
*Psychological well-being*	67.7 ± 9.2	70.0

SD, standard deviation.

Table 2 shows that for men and women, predictors such as age, employment status, educational level, religious beliefs, financial dependence, self-reported health, community participation, time spent with friends, daily internet use, and family relationship score were all associated with PWB with *p-*values and confidence intervals indicating significant effects. Satisfaction with living environment and place of residence were significantly associated with women but not with men. On the other hand, marital status, financial stress, and chronic diseases were significantly associated with PWB in men but not women as indicated by the significant p values and confidence intervals.

**Table 2:** Associations between predictors and psychological well-being among older Taiwanese men and women.

	Overall	Men	Women
Variable	Odds ratio (95% CI)	Odds ratio (95% CI)	Odds ratio (95% CI)
*Gender*			
Male	1.000		
Female	0.637 (0.514 ~ 0.790) ^**^		
*Age*	0.964 (0.949 ~ 0.980) ^**^	0.959 (0.937 ~ 0.981) ^**^	0.968 (0.948 ~ 0.988) ^**^
*Marital status*			
Not married	1.000	1.000	1.000
Married	1.484 (1.184 ~ 1.860) ^**^	2.485 (1.573 ~ 3.926) ^**^	1.062 (0.815 ~ 1.386)
*Employment*			
Unemployed	1.000	1.000	1.000
Employed	2.127 (1.574 ~ 2.875) ^**^	2.265 (1.714 ~ 4.019) ^**^	1.532 (1.006 ~ 2.333) ^*^
*Educational level*			
None	1.000	1.000	1.000
Primary	1.953 (1.460 ~ 2.612) ^**^	1.304 (0.750 ~ 2.268)	2.096 (1.522 ~ 2.888) ^**^
Secondary	3.577 (2.559 ~ 5.000) ^**^	2.528 (1.412 ~ 4.528) ^**^	3.897 (2.561 ~ 5.932) ^**^
Tertiary	8.074 (4.916 ~ 13.262) ^**^	6.169 (2.986 ~ 12.743) ^**^	8.517(3.852 ~ 18.831) ^**^
*Religious beliefs*			
No	1.000	1.000	1.000
Yes	1.385 (1.034 ~ 1.855) ^**^	1.426 (0.952 ~ 2.138)	1.707 (1.140 ~ 2.556) ^**^
*Financial stress*			
High	1.000	1.000	1.000
Low	2.043 (1.566 ~ 2.667) ^**^	2.251 (1.531 ~ 3.309) ^**^	2.010 (1.453 ~ 2.781)
*Satisfaction with the living environment*			
Dissatisfied	1.000	1.000	1.000
Satisfied	1.385 (1.104 ~ 1.737) ^**^	1.375 (0.971 ~ 1.945)	1.413 (1.044 ~ 1.913) ^**^
*Place of residence*			
Metropolitan	1.000	1.000	1.000
Other	0.643 (0.511 ~ 0.811) ^**^	0.699 (0.487 ~ 1.004)	0.604(0.446 ~ 0.817) ^**^
*Financial dependence*			
Not dependent	1.000	1.000	1.000
Dependent	0.402 (0.324 ~ 0.499) ^**^	0.375 (0.272 ~ 0.518) ^**^	0.475 (0.350 ~ 0.645) ^**^
*Caregiver role*			
No	1.000	1.000	1.000
Yes	0.774 (0.584 ~ 1.027)	0.909 (0.579 ~ 1.426)	0.714 (0.494 ~ 1.033)
*Self-reported health*			
Poor	1.000	1.000	1.000
Fair	2.778 (2.085 ~ 3.702) ^**^	2.833 (1.806 ~ 4.445) ^**^	2.707 (1.862 ~ 3.936) ^**^
Good	5.521 (4.4083 ~ 7.466) ^**^	5.997 (3.738 ~ 9.619) ^**^	5.056 (3.409 ~ 7.501) ^**^
*Chronic diseases*			
No	1.000	1.000	1.000
Yes	0.709 (0.572 ~ 0.879) ^**^	0.636 (0.458 ~ 0.884) ^**^	0.807 (0.604 ~ 1.076)
*Sleep Quality*			
Poor	1.000	1.000	1.000
Fair	1.331 (1.008 ~ 1.756) ^*^	0.899 (0.566 ~ 1.427)	1.649 (1.162 ~ 2.340)
Good	2.169 (1.686 ~ 2.790) ^**^	1.798 (1.206 ~ 2.682) ^**^	2.212 (1.587 ~ 3.082) ^**^
*Body pain*			
None	1.000	1.000	1.000
Mild to moderate	0.590 (0.478 ~ 0.729) ^**^	0.598 (0.433 ~ 0.824) ^**^	0.642 (0.482 ~ 0.854) ^**^
Severe	0.235 (0.141 ~ 0.392) ^**^	0.217 (0.084 ~ 0.557) ^**^	0.274 (0.148 ~ 0.507) ^**^
*Community participation*			
No	1.000	1.000	1.000
Yes	2.806 (2.248 ~ 3.502) ^**^	3.954 (2.814 ~ 5.555) ^**^	2.331 (1.769 ~ 3.071) ^**^
*Time spent with friends*			
Little	1.000	1.000	1.000
Fair	2.422 (1.921 ~ 3.054) ^**^	2.667 (1.901 ~ 3.742) ^**^	2.400 (1.788 ~ 3.223) ^**^
A lot	2.458 (1.579 ~ 3.826) ^**^	3.561 (1.787 ~ 9.095) ^**^	2.107 (1.225 ~ 3.624) ^**^
*Daily internet use*			
No	1.000	1.000	1.000
Yes	3.429 (2.565 ~ 4.583) ^**^	3.056 (2.005 ~ 4.657) ^**^	3.561 (2.380 ~ 5.328) ^**^
*Family relationship score*			
Low	1.000	1.000	1.000
High	2.544 (2.012 ~ 3.215) ^**^	2.670 (1.883 ~ 3.785) ^**^	2.587 (1.876 ~ 3.569) ^**^

*p < 0.05, ^**^p < 0.01. CI, confidence interval.

Table 3 illustrates that when controlled, several socioeconomic, health, behavioral, and social-related predictors were observed to be significantly associated with PWB. These included: employment status (OR = 1.648, 95% CI [1.135–2.394], *p* < 0.01), educational level (primary: OR = 1.698, 95% CI [1.215–2.373], *p* < 0.01; secondary: OR = 2.188, 95% CI [1.444–3.315], *p* < 0.01; tertiary: OR = 4.487, 95% CI [2.415–8.337], *p* < 0.01), religious beliefs (OR = 1.806, 95% CI [1.281–2.545], *p* < 0.01), satisfaction with living environment (OR = 1.486, 95% CI [1.117–1.976], *p* < 0.01), financial dependence (OR = 0.705, 95% CI [0.526–0.946], *p* < 0.05), self-reported health (fair: OR = 1.803, 95% CI [1.255–2.591], *p* < 0.01; good: OR = 2.657, 95% CI [1.786–3.953], *p* < 0.01), chronic diseases (OR = 0.738, 95% CI [0.557–0.977], *p* < 0.05), community participation (OR = 2.044, 95% CI [1.589–2.629], *p* < 0.01), time spent with friends (fair: OR = 2.078, 95% CI [1.591–2.714], *p* < 0.01; a lot: OR = 2.304, 95% CI [1.406–3.777], *p* < 0.01), daily internet use (OR = 1.599, 95% CI [1.098–2.327], *p* < 0.01), and family relationship score (OR = 1.988, 95% CI [1.494–2.646], *p* < 0.01).

**Table 3:** Multiple logistic regression analysis of predictors of psychological well-being among older Taiwanese men and women.

	Overall	Men	Women
Variable	Odds ratio (95% CI)	Odds ratio (95% CI)	Odds ratio (95% CI)
*Gender*			
Male	1.000		
Female	0.885 (0.665 ~ 1.178)		
*Age*	1.007 (0.987 ~ 1.028)	1.018 (0.986 ~ 1.051)	1.001 (0.974 ~ 1.030)
*Marital status*			
Not married	1.000	1.000	1.000
Married	0.891 (0.662 ~ 1.200)	1.584 (0.863 ~ 2.908)	0.725 (0.507 ~ 1.038)
*Employment*			
Unemployed	1.000	1.000	1.000
Employed	1.648 (1.135 ~ 2.394) ^**^	2.097 (1.196 ~ 3.677) ^*^	1.463 (0.862 ~ 2.482)
*Educational level*			
None	1.000	1.000	1.000
Primary	1.698 (1.215 ~ 2.373) ^**^	0.891 (0.438 ~ 1.812)	2.029 (1.374 ~ 2.995) ^**^
Secondary	2.188 (1.444 ~ 3.315) ^**^	1.301 (0.605 ~ 2.795)	2.475 (1.440 ~ 4.257) ^**^
Tertiary	4.487 (2.415 ~ 8.337) ^**^	2.755 (1.082 ~ 7.018) ^*^	4.904 (1.860 ~ 12.928) ^**^
*Religious beliefs*			
None	1.000	1.000	1.000
Yes	1.806 (1.281 ~ 2.545) ^**^	1.753 (1.043 ~ 2.994) ^*^	1.909 (1.188 ~ 3.3068) ^**^
*Financial stress*			
High	1.000	1.000	1.000
Low	1.210 (0.886 ~ 1.652)	1.459 (0.889 ~ 2.395)	1.007 (0.667 ~ 1.521)
*Satisfaction with the living environment*			
Unsatisfied	1.000	1.000	1.000
Satisfied	1.486 (1.117 ~ 1.976) ^**^	1.369 (0.874 ~ 2.145)	1.566 (1.063 ~ 2.305) ^*^
*Place of residence*			
Metropolitan	1.000	1.000	1.000
Other	0.613 (0.462 ~ 0.812) ^**^	0.566 (0.360 ~ 0.891) ^*^	0.638 (0.439 ~ 0.926) ^*^
*Financial dependence*			
Not dependent	1.000	1.000	1.000
Dependent	0.705 (0.526 ~ 0.946) ^*^	0.697(0.448 ~ 1.086) ^*^	0.673 (0.448 ~ 1.012)
*Caregiver role*			
No	1.000	1.000	1.000
Yes	0.885 (0.627 ~ 1.250)	0.982 (0.557 ~ 1.731)	0.850 (0.540 ~ 1.337)
*Self-reported health*			
Poor	1.000	1.000	1.000
Fair	1.803 (1.255 ~ 2.591) ^**^	1.925 (1.078 ~ 3.437) ^*^	1.747 (1.078 ~ 2.830) ^*^
Good	2.657 (1.786 ~ 3.953) ^**^	2.612 (1.394 ~ 4.893) ^**^	2.710 (1.590 ~ 4.619) ^**^
*Chronic diseases*			
No	1.000	1.000	1.000
Yes	0.738 (0.557 ~ 0.977) ^*^	0.548 (0.355 ~ 0.846) ^*^	0.919 (0.625 ~ 1.351)
*Sleep Quality*			
Poor	1.000	1.000	1.000
Fair	0.836 (0.591 ~ 1.182)	0.548 (0.300 ~ 1.002)	1.059 (0.685 ~ 1.636)
Good	0.984 (0.713 ~ 1.357)	0.745 (0.437 ~ 1.273)	1.166 (0.768 ~ 1.770)
*Body pain*			
None	1.000	1.000	1.000
Mild to moderate	0.787 (0.603 ~ 1.026)	0.733 (0.481 ~ 1.116)	0.794 (0.558 ~ 1.130)
Severe	0.534 (0.265 ~ 1.077)	0.855 (0.231 ~ 3.161)	0.445 (0.185 ~ 1.070)
*Community participation*			
No	1.000	1.000	1.000
Yes	2.044 (1.589 ~ 2.629) ^**^	2.763 (1.833 ~ 4.165) ^**^	1.717 (1.233 ~ 2.392) ^**^
*Time spent with friends*			
Little	1.000	1.000	1.000
Fair	2.078 (1.591 ~ 2.714) ^**^	2.548 (1.666 ~ 3.897) ^**^	1.764 (1.239 ~ 2.510) ^**^
A lot	2.304 (1.406 ~ 3.777) ^**^	4.528 (1.888 ~ 10.861) ^**^	1.496 (0.795 ~ 2.815)
*Daily internet use*			
No	1.000	1.000	1.000
Yes	1.599 (1.098 ~ 2.327) ^**^	1.615 (0.929 ~ 2.808)	1.712 (1.009 ~ 2.905) ^*^
*Family relationship score*			
Low	1.000	1.000	1.000
High	1.988 (1.494 ~ 2.646) ^**^	1.466 (0.923 ~ 2.328)	2.446 (1.659 ~ 3.605) ^**^

*p < 0.05, ^**^p < 0.01. CI, confidence interval.

However, it was observed that the financial stress, caregiver role, sleep quality, and body pain predictors did not show significant associations with PWB when controlled.

Table 3 also illustrates that when gender differences were examined the predictors had varying effects on PWB when controlled. Notably, most of these predictors were socioeconomic in nature. There was a stronger and more significant association observed among men (men: OR = 2.097, 95% CI [1.196–3.677], *p* < 0.05; women: OR = 1.463, 95% CI [0.862–2.482], *p* > 0.05). Higher education was associated with increased odds of PWB for both genders however, this was particularly pronounced for women. For women, all levels of education increased the odds for PWB (primary: women OR = 2.029, 95% CI [1.374–2.995], *p* < 0.01; secondary: women OR = 2.475, 95% CI [1.440–4.257], p < 0.01; tertiary: women OR = 4.904, 95% CI [1.860–12.928], *p* < 0.01). Chronic diseases were found to be associated with lower odds of PWB for men (OR = 0.548, 95% CI [0.355–0.846], *p* < 0.05), but there was no significant association among women (OR = 0.919, 95% CI [0.625–1.351], *p* > 0.05). Higher family relationship scores remained significantly associated with PWB among women (OR = 2.446, 95% CI [1.659–3.605], *p* < 0.01), but not among men (OR = 1.466, 95% CI [0.923–2.328], *p* > 0.05).

## Discussion

The relationship between gender and PWB was previously described as unclear (Matud et al., 2019). This study addressed this research gap among older populations and investigated how socioeconomic, health, behavioral, and social factors predict PWB and how gender differences influence these associations.

### Socioeconomic predictors of psychological well-being and gender differences

In contrast to other studies, marriage was not found to be a significant predictor of PWB when controlled for other socio-economic, health, social, and behavioral factors. It can then be suggested that marriage quality or marriage happiness would be a better predictor of PWB among older Taiwanese adults.

Son preference is a cultural phenomenon that was dominant in pre-industrialized Taiwan before the 1960s (Lin, 2009). This preference for male children meant that boys were more educated than girls, facilitating resource substitution. Resource substitution is a theory that states that education improves the PWB of women more than men due to inequity in socioeconomics. This postulates that women depend more on education to achieve well-being than their male counterparts (Ross and Mirowsky, 2006). Thus the effect of a single factor such as education is amplified in the marginalized group and is less significant in the preferred group (Ross and Mirowsky, 2010). This could account for the amplified effect education had on PWB for women in comparison to men. Education can potentially promote autonomy, positive relations with others, and personal growth, the foundations of psychological well-being.

The study’s negative associations of financial dependence and unemployment with PWB are consistent with what is in the literature (Ryff and Singer, 2008; Mousteri et al., 2018; Moxham et al., 2018; Pak, 2020). Distinctive to this study, these relationships were notably observed in men. Taiwan’s traditional Confucian values often depict men as providers and having a higher socioeconomic status than women (Lin, 2009); therefore, financial dependence and unemployment may negatively affect the PWB of men more than women in this cultural context. Older men unlike women are expected to support their families financially. If such expectations become aligned with a man’s sense of purpose and autonomy then when they are no longer able to work and became financially dependent, their well-being could be diminished.

It is believed that religion provides positive intrapersonal factors, e.g., hope, and interpersonal factors, e.g., social interactions with others improve mental health (Joshi et al., 2008). Religion can also promote happiness and a sense of purpose which are key in assessing PWB. This accounts for religion being a strong predictor of PWB in both men and women (Li, 2014).

Unsatisfactory physical living conditions could be a source of contention within a family. This could potentially be internalized more by women who may feel more burdened to maintain harmony in the home due to traditional Confucian values (Chao and Roth, 2000). Additionally, women tend to be more sensitive to environmental security issues than men (Bentes et al., 2017). This highlights potential directions for further research.

Metropolitan regions had a notably more positive influence on the PWB of older Taiwanese adults irrespective of gender highlighting their advantages over their limitations. Happiness in older populations is associated with the availability of good-quality services such as transportation (Krefis et al., 2018). Additionally, the presence of green and blue spaces, both of which are popular in Taiwan’s metropolitan areas have been found to promote good mental well-being (Krefis et al., 2018). Older adults in rural regions often encounter limited access to adequate infrastructure. Additionally, they may experience empty nests, as their younger family members migrate to the city to improve their livelihoods, leaving them without caretakers (Srivastava, 2009). This may result in loneliness and feelings of abandonment, potentially contributing to psychological distress.

### Health predictors of psychological well-being and gender differences

Self-rated health (SRH) reflects one’s perception of their health while the presence of chronic disease reflects a medically diagnosed illness. Distinctive to previous studies, this study examines gender differences in the perception of good health and a medically diagnosed chronic disease and its impact on PWB. Consistent with the literature, (Williams et al., 2017) as higher SRH significantly improved PWB outcomes in men and women. However, the relationship between the presence of chronic disease and PWB was only significant for men. It can be suggested that when controlled for SRH, chronic disease is not a significant predictor of PWB for women as they are more aware of their health status and manage chronic diseases more efficiently. Thus, reducing potential effects on PWB. Men tend to be more reluctant to seek and articulate health issues to avoid the perception of being “weak” (Matud, 2017). A medical diagnosis forces men to confront their ill health, potentially contributing to psychological distress. Thus, it can be deduced that the inclusion of medically diagnosed conditions is particularly useful when examining outcomes for men.

### Social predictors of psychological well-being and gender differences

The results of this study were consistent with the literature as good family relationships and friendships were positively associated with higher PWB (Tramonti et al., 2019). Taiwanese culture exhibits strong familial values (Chiao et al., 2019); however, this association was only observed in women in this study. As women are traditionally tasked with the responsibility of maintaining harmony and cohesion within the family (Yang, 2016), thus, older Taiwanese women may be more sensitive than men to difficulties and dysfunction within the family. Consequently, positive family relationships may have a more positive influence on their PWB compared to men. One can suggest that women might place greater emphasis on family relationships, which could explain why there was a significant association between the variable “spending a fair amount of time with friends” and not “spending a lot of time with friends.” Friendships play a role in higher PWB among older adults (Thanakwang et al., 2012; Greenfield and Reyes, 2014) as they promote social support and pleasant experiences as aging progresses (Ng et al., 2021). From this study, spending time with friends appeared to be a better predictor of PWB for women than men, while women’s PWB is more heavily influenced by family relationships.

### Behavioral predictors of psychological well-being and gender differences

Consistent with the literature, community participation promoted higher PWB in both men and women (Gyasi et al., 2019). Community participation is associated with increased social support, health and well-being (Jones et al., 2013). Community participation may be a suitable approach to PWB interventions aimed at capturing both male and female older Taiwanese adults.

Generally, the literature states that internet use is associated with PWB in both men and women (Quintana et al., 2018; Wang et al., 2019). It is difficult to ascertain why this type of association was significant for women but not for men without information. Finding out the purpose of internet use may give insight into these differences. As the world becomes more digital, it is worthwhile to deeply explore internet use among older populations and its impact on PWB.

## Conclusion

### Research questions and hypothesis

The findings of this study provide insight into the predictors of psychological well-being.

Overall, employment, education, religious beliefs, satisfaction with the living environment, living in a metropolitan region, financial dependence, self-rated health, chronic disease, community participation, time spent with friends, family relationship score, and daily internet use were all significant predictors of PWB. Financial dependence and chronic disease both distinctively negatively impact PWB outcomes.

Employment positively predicts PWB in men. Financial dependence and chronic disease both predict negative PWB in men. While education positively predicts PWB for both men and women, it has a greater impact on women than men. Satisfaction with the living environment was found to be a positive predictor for PWB in women. While spending time with friends positively predicts PWB for both men and women, it has less impact on women than men. Additionally, family relationships positively predicted PWB in women but had no significant impact on men.

In conclusion, this study supports the hypothesis. There are gender differences in the socioeconomic, health, social, and behavioral predictors of psychological well-being among older Taiwanese adults.

### Implications for public policy

Several interventions could be considered to improve the psychological well-being of older Taiwanese adults. Educational Programs targeted at older adults could be beneficial as education is a strong predictor of PWB for women and financial dependence is a strong predictor of PWB for men. Programs focused on vocational training or skill development may help empower them, increasing their sense of purpose and self-esteem. Such programs can also encourage older adults to create other sources of income. Separate educational programs can be specifically tailored to suit the needs of older Taiwanese men and women.

Social Support Workshops targeted at older adults could also be beneficial. These workshops could be aimed at strengthening and improving family dynamics as family relationships are a strong predictor of PWB for women. These workshops can involve counseling services surrounding mental and physiological health, especially for men as the diagnosis of a chronic disease is a strong predictor of lower PWB outcomes for men. This can be facilitated by community and religious groups as well as healthcare institutions.

Community engagement initiatives could also be beneficial to PWB in both men and women. Such initiatives could include social activities, cultural activities, and volunteer opportunities. This will promote social connections and a sense of belonging and purpose. Community health-based programs can provide opportunities for screening for chronic diseases and assistance with disease management.

Urban Planning and Infrastructure Development could be beneficial in improving PWB in older adults. Initiatives aimed at creating age-friendly environments such as accessible mental health services for older adults with chronic conditions, accessible public spaces, reliable and frequent public transportation services, and accessible recreation will promote active aging and social inclusion, especially outside the metropolitan centers.

### Limitations

This study is cross-sectional thus causation cannot be determined. Additionally, PWB can be affected by recent positive or negative events, thus the recorded PWB may be different from the participant’s norm.

Variables were self-reported which might be susceptible to recall and social desirability bias. Variables such as financial stress, satisfaction with the living environment, and time spent with friends were subjective. Objective tools of measurement of these variables would improve the robustness of the data.

The dataset was fixed which limited scope. Therefore, new hypotheses and research questions that arose during data analysis could not be explored nor could additional relevant variables be considered. Additionally, individuals without household registration and those institutionalized in retirement homes were excluded thus limiting generalizability.

While the results of this study share several similarities with the results of other studies, the influence of the cultural context of Taiwan must be considered. This can reduce the extent to which it can be applied globally in other populations.

### Recommendations

It is highly recommended that this study be replicated through several longitudinal studies focusing on gender-specific analyses of predictors of PWB. Specifically, future research should explore detailed examinations of predictors such as marital happiness rather than marital status, place of residence and the living environment, family dynamics, chronic health conditions, and financial dependency.

It is also recommended that intervention studies be conducted to evaluate the effectiveness of education programs, urban planning and infrastructure development, and the establishment of social support networks.

## Data availability statement

The raw data supporting the conclusions of this article will be made available by the authors, without undue reservation.

## Ethics statement

The studies involving humans were approved by Taipei Medical University Joint Institutional Review Board (no.: 201608036). The studies were conducted in accordance with the local legislation and institutional requirements. The participants provided their written informed consent to participate in this study.

## Author contributions

DF: Writing – review & editing, Writing – original draft, Formal analysis, Data curation. Y-HC: Writing – review & editing, Writing – original draft, Methodology, Conceptualization. Y-CC: Writing – review & editing, Writing – original draft, Methodology, Conceptualization. A-HM: Writing – review & editing, Writing – original draft, Methodology, Conceptualization. K-YC: Writing – review & editing, Writing – original draft, Methodology, Formal analysis, Conceptualization.
